# Late progamic phase and fertilization affect calreticulin expression in the *Hyacinthus orientalis* female gametophyte

**DOI:** 10.1007/s00299-015-1863-0

**Published:** 2015-09-09

**Authors:** Katarzyna Niedojadło, Robert Lenartowski, Marta Lenartowska, Elżbieta Bednarska-Kozakiewicz

**Affiliations:** Department of Cell Biology, Faculty of Biology and Environment Protection, Nicolaus Copernicus University in Toruń, Toruń, Poland; Laboratory of Isotope and Instrumental Analysis, Faculty of Biology and Environment Protection, Nicolaus Copernicus University in Toruń, Toruń, Poland; Laboratory of Developmental Biology, Faculty of Biology and Environment Protection, Nicolaus Copernicus University in Toruń, Toruń, Poland

**Keywords:** Ca^2+^ homeostasis, Calreticulin, Embryo sac, Egg apparatus, Fertilization, Filiform apparatus

## Abstract

*****Key message***:**

**Calreticulin expression is upregulated during sexual reproduction of*Hyacinthus orientalis*, and the protein is localized both in the cytoplasm and a highly specialized cell wall within the female gametophyte.**

**Abstract:**

Several evidences indicate calreticulin (CRT) as an important calcium (Ca^2+^)-binding protein that is involved in the generative reproduction of higher plants, including both pre-fertilization and post-fertilization events. Because CRT is able to bind and sequester exchangeable Ca^2+^, it can serve as a mobile intracellular store of easily releasable Ca^2+^ and control its local cytosolic concentrations in the embryo sac. This phenomenon seems to be essential during the late progamic phase, gamete fusion, and early embryogenesis. In this report, we demonstrate the differential expression of CRT within *Hyacinthus* female gametophyte cells before and during anthesis, during the late progamic phase when the pollen tube enters the embryo sac, and at the moment of fertilization and zygote/early endosperm activation. CRT mRNA and the protein localize mainly to the endoplasmic reticulum (ER) and Golgi compartments of the cells, which are involved in sexual reproduction events, such as those in sister synergids, the egg cell, the central cell, zygote and the developing endosperm. Additionally, immunogold research demonstrates selective CRT distribution in the filiform apparatus (FA), a highly specific component of the synergid cell wall. In the light of our previous data showing the total transcriptional activity of the *Hyacinthus* female gametophyte and the results presented here, we discuss the possible functions of CRT with respect to the critical role of Ca^2+^ homeostasis during key events of sexual plant reproduction. Moreover, we propose that the elevated expression of CRT within the female gametophyte is a universal phenomenon in the cells involved in double fertilization in higher plants.

## Introduction

CRT is an abundant Ca^2+^-binding/buffering protein that is predominantly localized in the ER of eukaryotic cells, where it acts as a lectin-like chaperone that is involved in the proper folding and quality control of de novo-synthesized proteins and is a modulator of Ca^2+^ homeostasis and the signaling network (see reviews by Gelebart et al. 2005; Jia et al. 2009; Michalak et al. 2009; Thelin et al. 2011; Li and Yang 2012; Wang et al. 2012). This protein is composed of three distinct structural domains: an extremely conserved globular N-domain with an ER-targeting signal; a proline-rich P-domain that is responsible for chaperone activity and for binding Ca^2+^ with high affinity/low capacity; and a C-terminal domain that binds Ca^2+^ with a high capacity/low affinity that ends with a specific K/HDEL signal that is required for protein retention in the ER lumen (see reviews by Gelebart et al. 2005; Jia et al. 2009; Michalak et al. 2009; Thelin et al. 2011; Li and Yang 2012; Wang et al. 2012). Plant CRT shares similar structural organization and basic functioning with its animal homolog, and a wide range of developmental and environmental stimuli differentially affect CRT expression in plant cells (see reviews by Jia et al. 2009; Thelin et al. 2011; Li and Yang 2012; Qiu et al. 2012). However, current knowledge on the relevance of this protein to plant physiology is still very limited.

CRT retention in the ER lumen depends on the C-terminal signal, and all plant CRTs that have been cloned thus far contain the HDEL sequence. The protein has been detected in COPI-coated vesicles, confirming that an efficient mechanism for the retrieval of CRT from the Golgi compartment functions in plant cells (Pimpl and Denecke 2000). However, plant CRT can also become competent for export from the ER, as its isoform without the HDEL motif is transported via a COPII-dependent anterograde pathway (Phillipson et al. 2001). In fact, the localization of CRT outside of the ER has been frequently reported in plant cells, specifically in the Golgi stacks and secretory vesicles (Borisjuk et al. 1998; Navazio et al. 2002; Lenartowska et al. 2002, 2009; Hsieh and Huang 2005; Nardi et al. 2006; Lenartowski et al. 2015). CRT has also been localized in the cytosol (Lenartowska et al. 2002; Jia et al. 2008), protein bodies/protein storage vacuoles (Torres et al. 2001; Šamaj et al. 2008), nucleus and nuclear envelope (Denecke et al. 1995, Napier et al. 1995, Lenartowska et al. 2002; Lenartowski et al. 2015), and within the plasmodesmata (Baluška et al. 1999; Laporte et al. 2003; Chen et al. 2005; Lenartowska et al. 2009; Christensen et al. 2010; Demchenko et al. 2014). The presence of CRT was also confirmed in the plasma membrane and small patches adjacent to the plasma membrane as well as on the cell surface and even in the cell wall (Borisjuk et al. 1998; Lenartowska et al. 2002, 2009; Navazio et al. 2002; Luczak et al. 2014; Lenartowski et al. 2015). These findings suggest diverse roles for plant CRT in multiple cellular processes.

There are some indications that different CRT isoforms (CRTs) may be important during reproductive events in higher plants. The increased expression of the protein has been observed in barley ovaries 1 day after pollination and during the early stages of embryogenesis (Chen et al. 1994). Subsequently, similar data have been obtained in tobacco (Denecke et al. 1995), maize (Dresselhaus et al. 1996; Williams et al. 1997), *Ricinus* (Coughlan et al. 1997), *Arabidopsis* (Nelson et al. 1997; Christensen et al. 2010, Li et al. 2011), *Nicotiana* (Borisjuk et al. 1998), and *Petunia* (Lenartowski et al. 2014, 2015). It is unknown how CRT may participate in plant sexual reproduction, but it is obvious that Ca^2+^ signals control key biological functions, including double fertilization and development in plants (see reviews by Faure and Dumas 2001; Ge et al. 2007). The finding that a transient cytosolic Ca^2+^ increase triggers plant post-fertilization phases and corresponds to an upregulation of CRT expression suggests an important role for this protein in both pre-fertilization and post-fertilization events. The previous work on *Petunia* revealed that CRT is highly expressed within the female gametophyte of dicotyledonous plants in response to pollen tube arrival and fertilization (Lenartowski et al. 2014, 2015). It has been postulated that CRT may act as a Ca^2+^ buffer in regulating the cytosolic Ca^2+^ level during the late progamic phase, gamete fusion, and early embryogenesis. An important question is whether this high CRT expression is a universal phenomenon in cells that are involved in double fertilization. Thus, we examined the localization of CRT mRNA and protein within the embryo sac of the monocot *Hyacinthus,* and we discuss the functional role of CRT in the multi-step process of plant sexual reproduction.

## Materials and methods

### Plant material

Commercial cultivars of *Hyacinthus orientalis* L. were grown at room temperature. The *Hyacinthus* pistil is composed of a hollow style and a dry stigma, while the female gametophyte develops according to the *Polygonum* type and consists of two synergids, the egg cell, the central cell containing two polar nuclei, and three antipodals (Pięciński et al. 2008; Niedojadło et al. 2012a, b, 2015). For fluorescence and electron microscopy studies, the ovules were mechanically dissected from unpollinated flowers before and during anthesis and from hand-cross-pollinated flowers 8 h after pollination (at the late progamic phase when the pollen tubes have reached approximately three-quarters of the style length and have not entered the ovary) and 96 h after pollination (fertilized ovules). To examine pollen tube growth rates, pistils were dissected from pollinated flowers, cut along the longitudinal axis, stained with 0.1 % aniline blue according to the standard protocol, and observed by fluorescence microscopy (Olympus BX50). To verify the specificity of a primary rabbit-anti-CRT antibody (CRT PAb) from maize (Napier et al. 1995) by immunoblotting, whole pistils from unpollinated flowers of *Hyacinthus* and maize (as a positive control) were used.

### Sample processing

Dissected ovules were immediately fixed with freshly prepared 4 % formaldehyde (Polysciences) and 0.25 % glutaraldehyde (Sigma) in phosphate-buffered saline buffer (PBS), pH 7.2, for 24 h at 4 °C. For light microscopy studies, fixed ovules were dehydrated via a graded series of ethanol containing 10 mM dithiothreitol (DDT, Fermentas), supersaturated, and then embedded in BMM resin (butyl methacrylate, methyl methacrylate, 0.5 % benzoyl ethyl ether with 10 mM DDT, Fluka) at −20 °C under UV light for polymerization. For immunogold labeling, fixed and dehydrated ovules were embedded in LR Gold resin (Sigma). Polymerization with 1 % benzoyl peroxide as the accelerator occurred for 8 days at −20 °C.

Next, specimens were cut with glass or diamond knifes on a Leica UCT ultramicrotome into semithin or ultrathin sections. Semithin sections were placed on microscope slides that were covered with Biobond (British Biocell), while ultrathin sections were collected on nickel grids that were coated with 0.3 % Formvar (Sigma).

### Fluorescence in situ hybridization (FISH)

CRT mRNA was localized using an antisense digoxigenin (DIG)-UTP-labeled RNA probe that was generated by in vitro transcription using T7 polymerase following the manufacturer’s protocol (Roche). A maize CRT 1.6 kb cDNA clone (Napier et al. 1995) was used as the template to transcribe the probe that was used at a final concentration of 2.5 ng/μl. Pre-hybridization and hybridization were carried out in 50 % formamide, 4× SSC, 5× Denhart’s buffer, 1 mM EDTA, and 50 mM phosphate buffer. Hybridization was performed for 24 h at 42 °C. The signals were visualized after incubation with primary mouse-anti-DIG antibody (1:100, Roche) in PBS buffer, pH 7.2, supplemented with 0.01 % acetylated bovine serum albumin (acBSA, Sigma) for 12 h at 4 °C, followed by incubation with secondary goat-anti-mouse antibody conjugated with Alexa Fluor 488 (1:100, Invitrogen) in the same PBS buffer for 1 h at 37 °C. A no-probe control was also performed. In the final step, DNA was stained with 4,6-diamidino-2-phenylindole (DAPI, Fluka). Images were acquired using an Olympus BX50 fluorescence microscope. The UPlanFI 100× (N.A. 1.3) oil immersion lens and narrow band filters (U-MNU, U-MNG) were used. The results were registered with an Olympus XC50 digital color camera and Cell^B^ software (Olympus Soft Imaging Solutions GmbH, Münster, Germany).

### Immunolabeling

For light microscopy studies, after blocking with 3 % acBSA in PBS buffer, pH 7.2, for 0.5 h at room temperature, semithin sections were incubated with CRT PAb diluted 1:50 in PBS buffer, pH 7.2, with 0.01 % acBSA overnight at 4 °C. Signals were detected using secondary anti-rabbit IgG antibody that was conjugated with Cy3 (Sigma) diluted 1:1000 in the same PBS buffer with 0.01 % acBSA for 1 h at 37 °C. A control was performed without the primary antibody. In the final step, DNA was stained with DAPI (Fluka). Semithin sections were analyzed with an Olympus BX50 fluorescence microscope. UPlanFI 100× (N.A. 1.3) oil immersion lens and narrow band filters (U-MNU, U-MNG) were used. The results were registered with an Olympus XC50 digital color camera and Cell^B^ software (Olympus Soft Imaging Solutions GmbH, Münster, Germany).

For immunogold labeling, ultrathin sections were incubated with blocking solution containing 3 % acBSA in PBS buffer, pH 7.2, for 15 min at room temperature. Then, the sections were treated with 1:50 dilution of CRT PAb in the same PBS buffer supplemented with 0.2 % acBSA for 12 h at 4 °C. Antibody binding was detected by incubation with 15-nm-diameter gold-conjugated goat-anti-rabbit IgG antibody (BB International) diluted in PBS buffer (1:30) with 0.2 % acBSA for 1 h at 37 °C. Controls were performed by omitting incubation with the primary antibody. Finally, sections were stained with 1 % phosphotungstic acid and 5 % uranyl acetate solutions and examined using a JEOL 1010 transmission electron microscope at 80 kV.

### Western blot analysis

The specificity of the CRT PAb for hyacinth was verified by Western blot analysis according to a previously described protocol (Lenartowski et al. 2015). In brief, 100 mg of whole hyacinth and maize (positive control) pistils was powdered in liquid nitrogen, and the soluble proteins were extracted with 50 mM Tris–HCl (pH 7.5), 1 mM EGTA, 2 mM DTT plus 1 mM PMSF, and Complete Protease Inhibitor Cocktail (Roche). After centrifugation at 16,000 g for 30 min at 4 °C, the supernatants were collected, and the protein concentrations were determined by the Lowry method (Bio-Rad *DC* Protein Assay). Equal amounts of proteins (15 μg/lane) were separated by electrophoresis on a 12.0 % SDS-PAGE gel, transferred to Hybond-P membrane (GE Healthcare), and blocked with 2 % ECL Prime™ blocking reagent (GE Healthcare). The blots were probed with CRT PAb, washed, and then re-probed with anti-rabbit IgG antibody that was conjugated with horseradish peroxidase (HRP, Sigma). The final detection was performed with the Amersham ECL Advance Western Blotting Detection Kit according to the manufacturer’s guidelines (GE Healthcare).

### Quantitative analysis

Image analysis was performed on serial semithin sections after FISH, with each reaction step being performed using consistent values of temperature, incubation time, and probe concentration. The quantitative analysis of fluorescence spots was carried out for 5–7 of each cell type (5 sections per cell) from each development stage. All the measurements were conducted at the same magnification, field area (controlled with a shutter), and positioning of the fiber optics cable. The camera settings were kept constant for exposure time, gain and offset. Lucia G software was used to determine the average 100 µm^2^ number spots of fluorescence of each studied cell compartment. The obtained data were corrected for background autofluorescence as determined from negative control signal intensities. To test for differences among multiple samples, a Kruskal–Wallis ANOVA test was used. Statistical data and graphs were created using Microsoft Excel 2010 software.

## Results

### CRT is mainly expressed in the cells that are involved in double fertilization and in the zygote and developing endosperm

**Fig. 1:**
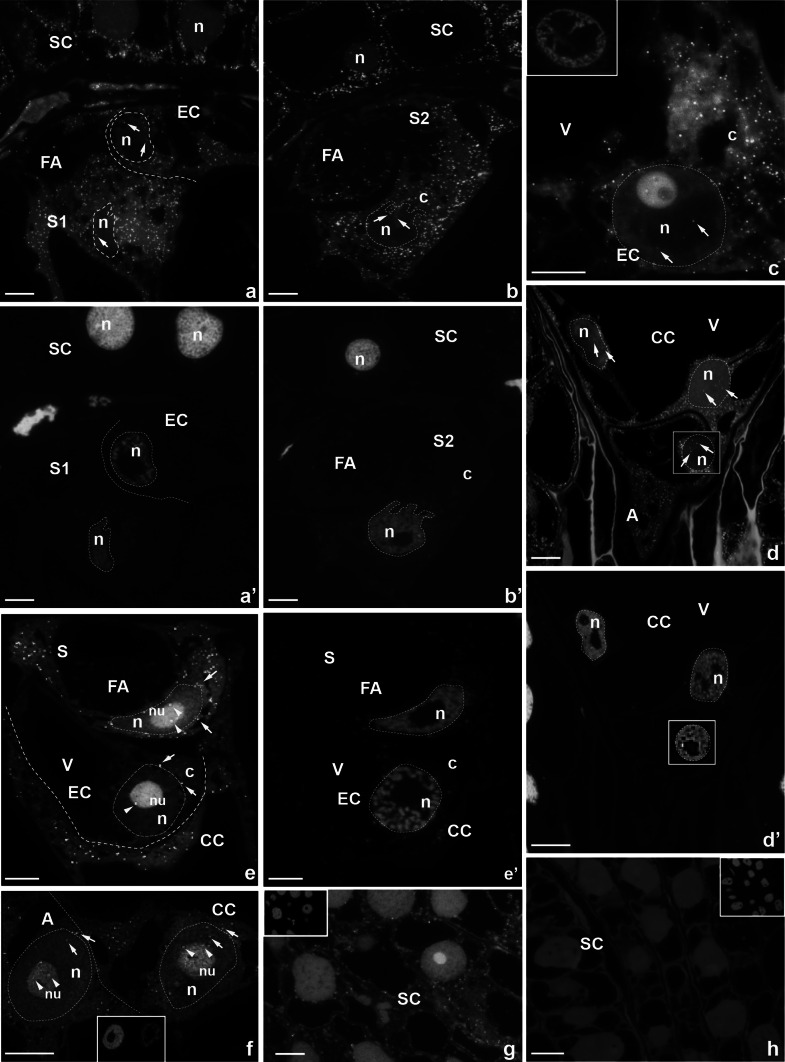
Localization of CRT mRNA transcripts in the 7-celled embryo sac of *H. orientalis* before (**a**–**d**) and during (**e**, **f**) anthesis. **a**–**c**, **e** micropylar end, **d**, **f** chalazal end (**d** the reconstruction of three serial sections; in the frame, the second polar nucleus and nucleus of antipodal cell are visible), **g** somatic cells, **h** no-probe control, **a′,** **b′,** **c′,** **d′,** **e′,** **f′,** **g′,** **h′** nuclei are stained with DAPI. *S* synergid cell, *FA* filiform apparatus, *EC* egg cell, *SC* somatic cell, *V* vacuole, *CC* central cell, *A* antipodal cell, *n* nucleus, *nu* nucleolus, *c* cytoplasm, *bars* 10 µm

**Fig. 2:**
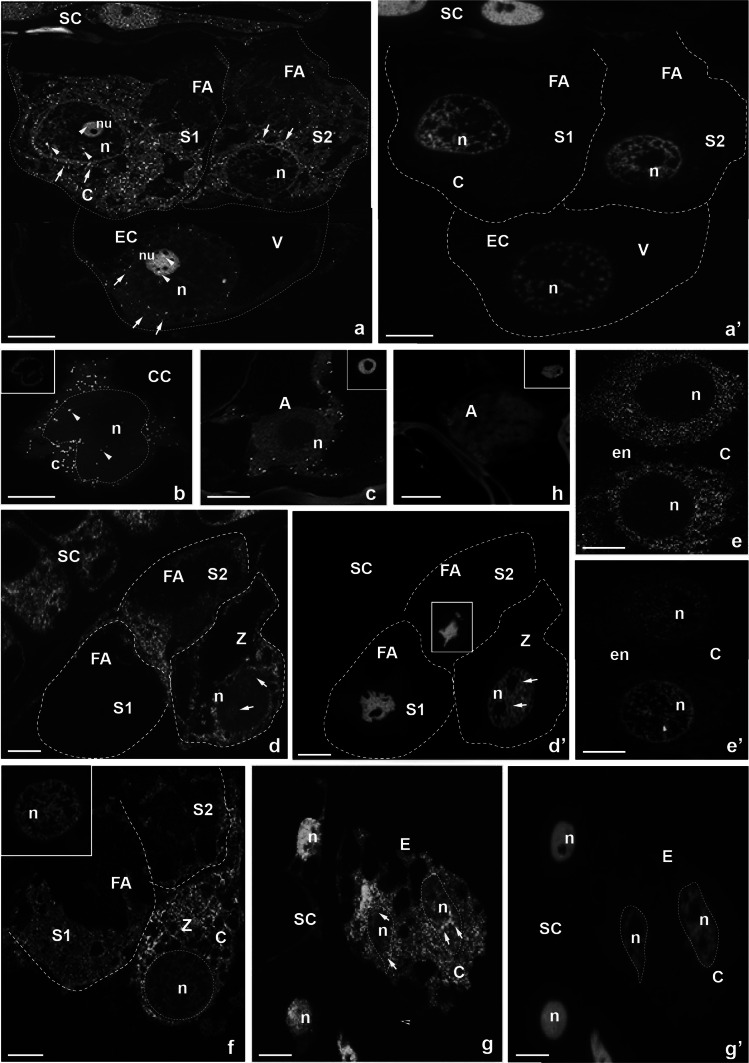
Localization of CRT mRNA transcripts in the *H. orientalis* embryo sac cells during the progamic phase (**a**–**c**) and after fertilization (**d**–**g**). **a** egg apparatus, **b** central cell, **c** antipodal cell, **d** micropylar end after fertilization, **e** first two nuclei of the endosperm, **f**, **g** embryo sac during the initiation of zygote division, **a′,** **b′,** **c′,** **d′,** **e′,** **f′,** **g′,** **h′** nuclei are stained with DAPI. *S* synergid cell, *FA* filiform apparatus, *EC* egg cell, *Z* zygote, *SC* somatic cell, *V* vacuole, *CC* central cell, *A* antipodal cell, *n* nucleus, *nu* nucleolus, *c* cytoplasm, *bars* 10 µm

The FISH technique clearly revealed an occurrence of CRT mRNA in genetically diverse cells of the female gametophyte and in the surrounding somatic cells of the ovule, from the cellularization period of the embryo sac until fertilization (Figs. 1, 2).

Before anthesis, at the micropylar pole of the mature embryo sac CRT transcripts were accumulated mainly in the cytoplasm of sister synergids (Fig. 1a, b), the egg cell (Fig. 1a, c), and the central cell (Fig. 1d). The presence of CRT mRNA in both synergids was primarily observed in the cytoplasm region below the FA, in which the hybridization signal was insignificant (Fig. 1a, b). In addition, in the nuclei of the egg apparatus cells (Fig. 1a–c) and in two polar nuclei of the central cell, localized to the chalazal end of the embryo sac at this developmental stage (Fig. 1d), fluorescent spots occurred occasionally (Fig. 1a–d, arrows). An analogous pattern of CRT transcripts distribution was observed in the antipodals (Fig. 1d) as well as in the somatic cells of the ovule adjacent to the embryo sac (Fig. 1a, b, d).

During anthesis, distribution pattern of CRT mRNA in the investigated cells was similar to that observed at the earlier developmental stage. The hybridization signal was still detected in the egg apparatus cells (Fig. 1e), in the central cell and antipodals (Fig. 1e, f), and in the somatic cells surrounding the embryo sac (Fig. 1g). The presence of CRT transcripts was still localized predominantly in the cytoplasm of investigated cells (Fig. 1e–g); however, few fluorescent spots were also observed around or within their nuclei (Fig. 1e, f, arrows) and nucleoli (Fig. 1e, f, arrowheads). Only the egg cell seems to show a reduced level of green fluorescence as compared to other cells (Fig. 1f). The negative control of FISH showed no green fluorescence in somatic cells, which were used as representatives (Fig. 1h).

Significant differences in the distribution pattern of CRT mRNA in the female gametophyte cells were observed at the late progamic phase, when pollen tubes were growing into the ovary and reached the ovules (Fig. 2a–c). The particular accumulation of CRT transcripts during this developmental stage was detected in one of the two sister synergids, in which the fluorescence signal was localized mainly in their cytoplasm, beyond the FA (Fig. 2a). The presence of investigated transcripts was also revealed in the second synergid cell (Fig. 2a) as well as in the central and antipodal cells (Fig. 2b, c, respectively). Only the egg cell showed a significantly lower level of green fluorescence, similar to that observed in the period prior to anthesis (Fig. 2a). As CRT transcripts were observed mainly in the cytoplasm of examined cells, including the area nearby their nuclei (Fig. 2a, arrows), a few spots of fluorescence were visible also in the nuclei and nucleoli (Fig. 2a, b arrowheads). Somatic cells surrounding the embryo sac still showed an accumulation of CRT mRNA (Fig. 2a).

A drastic change in the distribution of CRT mRNA in the female gametophyte cells was observed after fertilization. Soon after the gamete fusion, an accumulation of CRT transcripts was revealed in fertilized egg cell and in the primary endosperm (Fig. 2d, e, respectively), whereas sister synergids showed the still-diverse level of the fluorescence within their cytoplasm (Fig. 2d). In the zygote, in which one could observe a large central vacuole, the presence of the hybridization signal was also observed mainly in the cytoplasm (Fig. 2d). In contrast, in the nucleus of the zygote, in which two nucleoli were present, there were only single fluorescent spots (Fig. 2d, arrows). In the developing endosperm, a strong hybridization signal was predominantly reported in the whole cytoplasm (Fig. 2e). During the initial stage of embryogenesis, CRT mRNA was accumulated mainly in the dividing zygote (Fig. 2f) and in the developing endosperm (Fig. 2g). In the zygote, a strong hybridization signal was visible in the cytoplasm (Fig. 2f), whereas in the differentiating endosperm, numerous fluorescent spots were observed in both the cytoplasm (Fig. 2g) and in the nuclei (Fig. 2g, arrows). At the early embryogenesis stage, significant decrease of the fluorescence level was observed in sister synergids (Fig. 2f) and in degenerating antipodal cells (Fig. 2h), while in the somatic cells surrounding the embryo sac, an accumulation of CRT transcripts in the cytoplasm (Fig. 2d) and nuclei occurred (Fig. 2g).

**Fig. 3:**
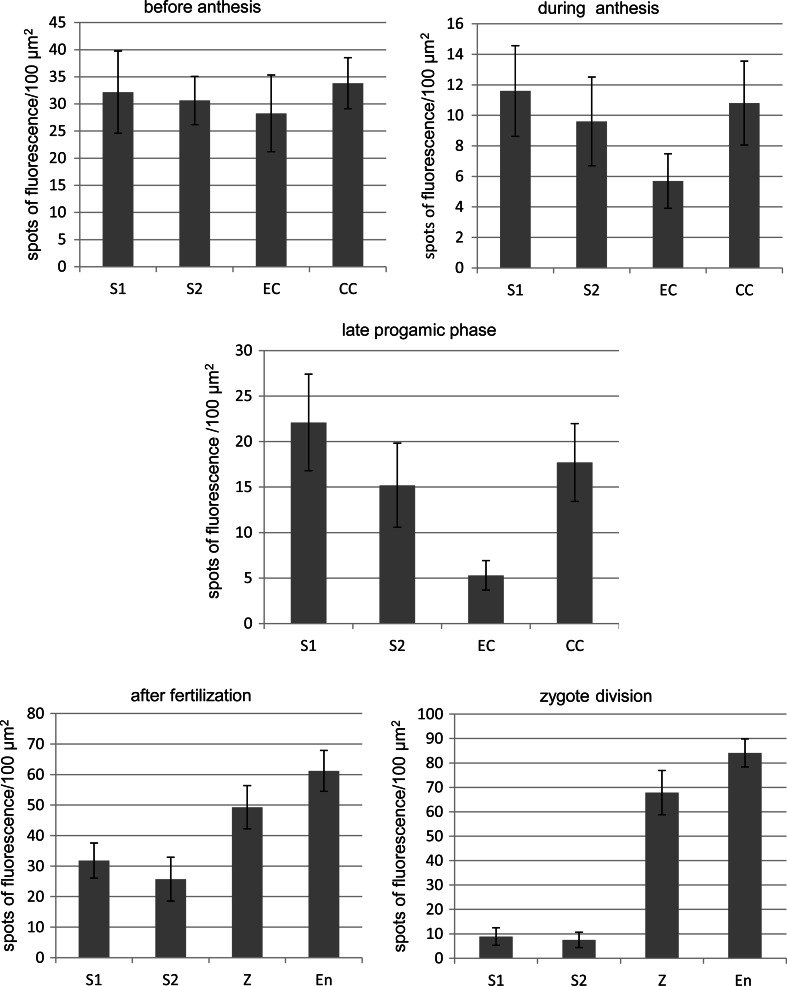
Histograms illustrating changes in the relative intensities of CRT mRNA transcript fluorescence (average 100 µm^2^ number spots of fluorescence) in *H. orientalis* embryo sac cells before and after fertilization. The quantitative analysis of fluorescence spots was carried out for 5–7 of each cell type (5 sections per cell) from each development stage. *Error bars* represent the standard deviation of the mean

Visualization by FISH provided an approximate measurement of the level of CRT transcripts that were evaluated based on the number of fluorescent spots per 100 um^2^ of the cell cytoplasm outside of the vacuole. Quantification analysis was applied to the cells of the egg apparatus, central cell, and then zygote and developing endosperm (Fig. 3). Before anthesis, the comparable level of CRT transcripts has been confirmed in the cells involved in double fertilization (Fig. 3a), whereas at anthesis a slightly lower level of CRT mRNA showed the egg cell (Fig. 3b). Interestingly, in sister synergids little difference in the quantity of CRT mRNA was reported after the flower opening (Fig. 3b). During the late progamic phase, the highest accumulation of investigated transcripts was detected in one of the two sister synergids and definitely lowest level of the fluorescence showed the egg cell (Fig. 3c). In contrast, after fertilization an accumulation of CRT mRNA showed the zygote and the developing endosperm, while progressive decrease in the level of fluorescence was observed in both synergids (Fig. 3d, e).

### CRT is localized both within the cytoplasm and in the highly specialized extracellular matrix of female gametophyte cells before and after fertilization

The distribution of CRT in the cells of the female gametophyte was assessed using immunocytochemical techniques at the cellular and subcellular levels using a fluorochrome- or gold-conjugated secondary antibodies. Particular attention was paid to the cells that were involved in the process of double fertilization (the egg apparatus and the central cell) and to the zygote and the developing endosperm.

**Fig. 4:**
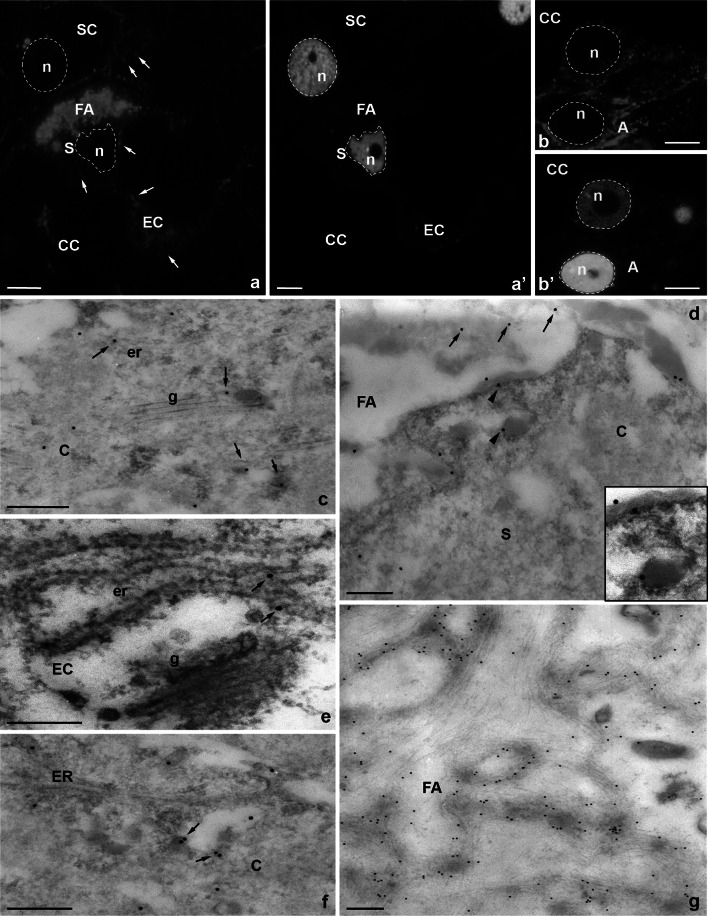
Immunofluorescence (**a**, **b**) and immunogold (**c**–**g**) localization of the CRT protein in *H. orientalis* embryo sac cells. **a**–**e** mature embryo sac, **a** micropylar end, **b** chalazal end, **c**, **d** synergid cell, **e** egg cell, **f**, **g** synergid cell during progamic phase, **a′**, **b′** nuclei are stained with DAPI. *S* synergid cell, *FA* filiform apparatus, *EC* egg cell, *CC* central cell, *SC* somatic cell, *A* antipodal cell, *n* nucleus, *c* cytoplasm, *er* endoplasmic reticulum, *g* Golgi apparatus, **a**, **b** *bars* 10 µm, **c**–**g** *bars* 200 nm

At the micropylar pole of the mature embryo sac, CRT was present in both synergids, in the egg cell, and in the somatic cells near the female gametophyte (Fig. 4a). However, the protein was occured mainly in the cytoplasm of these cells (Fig. 4a, arrows), its unique accumulation was also found in the FA of sister synergids (Fig. 4a). Immunofluorescence signal was also detected in the central cell and in the antipodals (Fig. 4b). Due to the unexpected occurence of CRT in the FA, distribution of this protein at the micropylar region of the embryo sac was also evaluated using the immunogold technique and electron microscopy (Fig. 4c, d). Subcellular studies have shown that, in synergids, CRT occurs primarily in the ER and dictyosomes (Fig. 4c) and in electron-opaque vesicles, which were located near the Golgi stacks (Fig. 4c, arrows). Moreover, the presence of CRT has been unambiguously confirmed in the FA of both synergid cells, wherein the gold traces were located at the edges of homogeneous electron-opaque regions (Fig. 4d, arrows) and in electron-dense vesicles that were located near the FA (Fig. 4d, arrowheads and inset). In addition, the location of CRT was shown in the cytoplasm of the egg cell in which the gold traces were associated mainly with the ER (Fig. 4e, arrows). A similar pattern of CRT distribution in synergids was observed during the progamic phase, in which the presence of the CRT was confirmed near the ER, including the electron-opaque vesicles (Fig. 4f, arrows) and in the area of electron-dense FA (Fig. 4g).

**Fig. 5:**
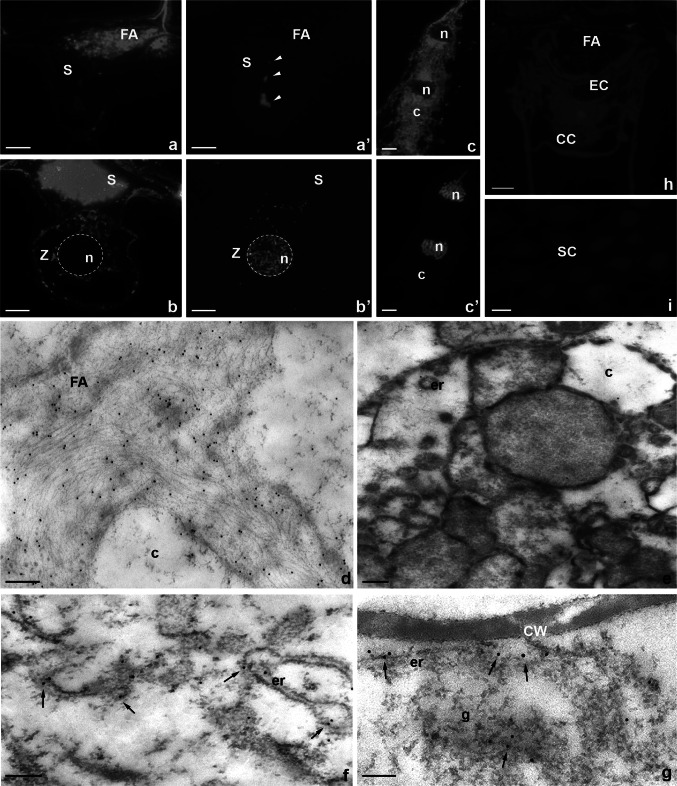
Immunofluorescence (**a**–**c**) and immunogold (**d**–**g**) localization of the CRT protein in *H. orientalis* embryo sac cells after fertilization. **a** receptive synergid cell, **b** persist synergid cell and the zygote, **c** the differentiating endosperm, **d** synergid cell, **e** the degenerating synergid cell, **f**, **g** zygote, **h**, **i** control reactions, **a′**, **b′**, **c′** nuclei are stained with DAPI. *S* synergid cell, *FA* filiform apparatus, *EC* egg cell, *CC* central cell, *SC* somatic cell, *n* nucleus, *c* cytoplasm, *er* endoplasmic reticulum, *g* Golgi apparatus, **a**–**c**, **h**, **i** *bars* 10 µm, **d**–**g** *bars* 200 nm

The CRT labeling pattern in the cells that were located in the micropylar region of the embryo sac after fertilization was similar. CRT was present in the synergids and the zygote as well (Fig. 5). The fluorescence was revealed in the receptive synergid (Fig. 5a), in which the cell nucleus and the nuclei of the released sperm cells were observed (Fig. 5a, arrowheads). CRT accumulation was also found in target cells for male gametes. In both the zygote (Fig. 5b) and the developing endosperm (Fig. 5c), the fluorescence was detected mainly in their cytoplasm. Studies using the electron microscopy have revealed that after fertilization, changes occur in the structure of the FA synergids, in which electro-opaque regions disappear with the simultaneous occurrence of the fibrous elements (Fig. 5d). Despite the differences that were observed in the structure of the FA, there were still numerous gold traces that were located in this area, along with long electron-dense fibrils (Fig. 5d). Subcellular studies have also revealed gold traces in the synergid cytoplasm after fertilization (Fig. 5e) and the presence of a cytoplasmic pool of CRT in the cytoplasm of the zygote, in which the protein occurred mainly in the ER (Fig. 5f, arrows). The cytoplasmic region of the zygote under the cell membrane was rich in the ER cisternae and dictyosomes that were specifically labeled by CRT PAb (Fig. 5g, arrows).

**Fig. 6:**
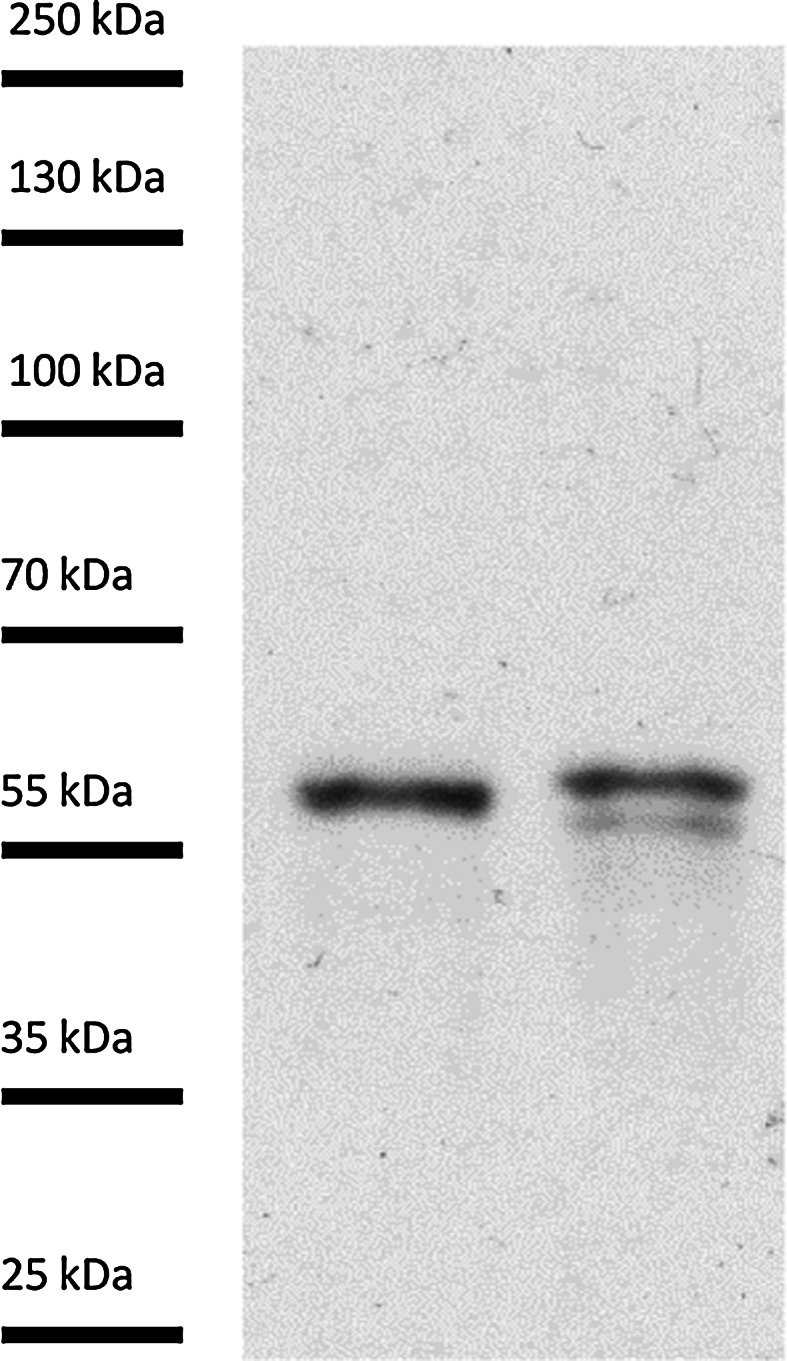
Western blotting of the total protein extracts using CRT PAb from *Zea mays* (*lane 1*) and *H. orientalis* (*lane 2*). The protein marker PageRuler Plus Prestained Protein Ladder (Life Technologies) is indicated on the *left*

The control sections in which no CRT PAb was used were devoid of labeling in the cells of the embryo sac (Fig. 5h) and in the somatic cells adjacent to the female gametophyte (Fig. 5i). The specificity of the primary CRT PAb from maize in the cells of hyacinth was tested by Western blot analysis (Fig. 6). While the antibody recognized a single band in extract made from maize (used as a positive control), a doublet was observed in *Hyacinthus* sample. This additional band may be a shorter isoform of CRT, as plants have been shown to contain two or more CRT isoforms with different weights corresponding to differences in N-linked glycosylation and/or phosphorylation. Alternatively, the smaller band could be a degradation product.

## Discussion

Here, we report for the first time the different expression of CRT at various stages of development/functioning of the female gametophyte in monocotyledonous plants, such as hyacinth. The results of our study indicate that prior to anthesis, all cells from the *Hyacinthus* embryo sac exhibit an accumulation of CRT mRNA, but significant differences in distribution pattern of investigated transcripts appear after flower opening, followed by the fusion of gametes. The observed changes in the expression of CRT in the cells that were involved in double fertilization are correlated in time with result previously reported by our group regarding the total transcriptional activity of *Hyacinthus* female gametophyte, whose level is comparable prior to anthesis but changes in response to flower opening, gamete fusion and early embryogenesis (Niedojadło et al. 2012a).

During the period preceding fertilization, from anthesis to the late progamic phase, the highest transcriptional activity of CRT was observed in synergids and in the central cell, while the metabolically silent egg cell exhibited reduced level of CRT expression (Niedojadło et al. 2012a and this work). Furthermore, during the progamic phase, there were clear differences in CRT expression in both synergids, in which mRNA CRT distribution is analogous to the location of incorporated 5′-bromouracil (BrU); the particular accumulation of these molecules occurs in the cytoplasm of sister synergids below the FA (Niedojadło et al. 2012a and this work). These observations are consistent with previous results that were obtained for dicotyledonous *Petunia* (Lenartowski et al. 2014) and indicate that the progamic phase is a period of increased transcriptional activity of CRT in cells whose participation in the adoption of the growing pollen tube is crucial (Kessler and Grossniklaus 2011; Bleckmann et al. 2014). One cannot exclude that the different CRT expression in sister synergids of two plant species belonging to different classes and different in terms of anatomical structure of pistil is universal elements of the cellular mechanism that determines the status of both synergids. This mechanism has still not been elucidated.

Our studies have also revealed the presence of CRT in all cells of the female gametophyte of hyacinth that participate in the process of sexual reproduction. In synergids, the egg cell and the central cell, in which the main locations of this protein in the cytoplasm are the ER and Golgi stacks, are recognized as the typical CRT localization sites and function as the mobile Ca^2+^ stores in plant cells (see reviews by Jia et al. 2009; Thelin et al. 2011; Stael et al. 2012). Particular attention in the period prior to fertilization should be paid to synergids, which play key roles in pollen tube guidance, followed by the inhibition of its growth and the opening of the tip, resulting in the release of sperm cells (Dresselhaus and Sprunck 2012; Leydon et al. 2014). During these processes, Ca^2+^-dependent signaling pathways and those that require precise control of the Ca^2+^ in the cytosol are required. Among all the cells of the embryo sac, synergids exhibit the highest level of loosely bound/stored/exchangeable Ca^2+^ (see review by Higashiyama 2002; Ge et al. 2007), while in several plant species, the unique accumulation of such a Ca^2+^ pool was observed in the receptive synergid (Denninger et al. 2014; Hamamura et al. 2014). Exchangeable Ca^2+^ is sequestered into different cell compartments (such as the ER), where it associates with specific proteins (such as CRT) that buffer and release Ca^2+^ to control its local cytosolic concentration. During the progamic phase in *Arabidopsis*, oscillations of ionic Ca^2+^ occurred in the cytosol of the receptive synergid that was transferred from the micropylar to the chalazal pole of the cell (Sandaklie-Nikolova et al. 2007; Iwano et al. 2012; Denninger et al. 2014; Ngo et al. 2014). In contrast, in the receptive synergid of *Petunia*, there was a specific micropylar–chalazal gradient of CRT mRNA and the protein at the time of sperm cell release (Lenartowski et al. 2014, 2015). Our current results demonstrate a significant increase in the expression level of CRT in the one of *Hyacinthus* synergids during the late progamic phase. Therefore, we propose that CRT is a universal key element of the mechanism that creates the specific Ca^2+^ oscillations and regulates the optimal Ca^2+^ environment within one of the synergids, possibly the receptive synergid that is probably essential during male gametes release and polyspermy block. The regulation of both of these phenomena at the molecular level is still in the realm of speculation (Denninger et al. 2014; Hamamura et al. 2014; Lenartowski et al. 2014, 2015).

Another significant change in the pattern of CRT expression in the cells of the *Hyacinthus* embryo sac occurs after the gamete fusion, when the increased transcriptional activation of zygotic and endosperm genome (Niedojadło et al. 2012a) is accompanied by an accumulation of CRT mRNA in the fertilized egg cell, the dividing zygote and the developing endosperm. In turn, in synergids degenerating after fertilization and antipodals, there is a silence of transcriptional activity, including CRT expression in these cells (Niedojadło et al. 2012a and this work). The results of our previous studies revealed that in hyacinth, the activation of the zygotic genome and the primary endosperm cell anticipates the first mitotic divisions over time, which confirms the high metabolic activity of a fertilized egg cell and the primary endosperm cell (Niedojadło et al. 2012a, b). In light of the current results, it seems highly likely that soon after karyogamy in the zygote, as well as in the primary endosperm, one also observes the upregulation of CRT expression. Our observations are confirmed by other authors who revealed a significant increase in the level of expression of CRT in response to the gamete fusion in the ovaries/ovules of different plant species. Elevated levels of CRT mRNA and/or CRT protein were observed at fertilization, rapid cell divisions of the zygote and the primary endosperm cell, and during early embryogenesis and endosperm development (Chen et al. 1994; Dresselhaus et al. 1996; Nelson et al. 1997; Borisjuk et al. 1998; Lenartowski et al. 2014, 2015). Moreover, in vitro fertilization initiates the occurrence of a Ca^2+^ wave in maize and tobacco zygote (Digonnet et al. 1997; Antoine et al. 2001; Peng et al. 2009). In turn, during the semi-in vivo fusion of gametes in *Arabidopsis*, there was a two-time occurrence of ionic Ca^2+^ peaks in the egg cell and central cell in the cytosol just after plasmogamy (Denninger et al. 2014; Hamamura et al. 2014). The observed Ca^2+^ signatures may result from the functioning of a hypothetical mechanism that uses mobile stores of exchangeable Ca^2+^ to regulate the level of ionic Ca^2+^ in the cytoplasm of target cells for male gametes. Because in hyacinth, similar to many other plant species, the presence of CRT mRNA and/or the CRT protein was confirmed in the zygote and in the developing endosperm, this protein is an ideal candidate to be a key component of the mechanism generating intracellular Ca^2+^ mobilization in response to fertilization. Moreover, the high level of CRT expression in the cytoplasm of the developing endosperm, which synthesizes nutrients for the differentiating embryo, may also be related to the intense protein synthesis during this developmental period. CRT controls the quality of newly synthesized glycoproteins that enter the ER before they are exported to various subcellular compartments (Michalak et al. 2009; Thelin et al. 2011; Qiu et al. 2012) and is also important for the normal trafficking of many surface proteins (Jiang et al. 2014). Therefore, some authors have suggested that CRT’s chaperone function may be requested for the proper folding of the proteins that are actively synthesized by the developing embryo and nutritive tissue (Chen et al. 1994; Dresselhaus et al. 1996; Nelson et al. 1997; Borisjuk et al. 1998, Lenartowski et al. 2014, 2015). The results of our study seem to confirm these assumptions.

One of the most interesting and surprising observations is the presence of CRT in the region of the FA of sister synergids. Before fertilization, we identified antigens that were conjugated to CRT PAb, which were localized to electron-opaque regions of the FA. Simultaneously, in the cytoplasm of synergids and in electron-opaque vesicles occurring near dictyosomes and under the cell membrane adjacent to the FA, we detected the presence of CRT in Golgi stacks. Interestingly, after fertilization, the FA ultrastructure in the *Hyacinthus* embryo sac changed; electron-opaque regions disappeared, and fibrillar structures appeared, with which CRT is still associated. Such locations of the CRT suggest that in synergids, the secretion pathway of this protein may operate from the ER via the Golgi stacks into the FA. The occurrence of CRT in extracellular regions is still controversial because it is not known how the protein containing the retention sequence for the ER diffuses beyond the cellular compartment. Several possible scenarios are taken into account, particularly the existence of CRT splice variants, which can leave the ER, or different posttranslational modifications of the protein (see reviews by Johnson et al. 2001; Michalak et al. 2009). However, an increasing evidence has indicated the occurrence of CRT on the cell surface or outside of the cell. In plants, CRT accumulation has been demonstrated in plasmodesmata (Baluška et al. 1999; Laporte et al. 2003; Bayer et al. 2004; Chen et al. 2005; Lenartowska et al. 2009; Christensen et al. 2010) and in the callosic cell wall of the pollen tube (Lenartowska et al. 2002, 2009). In contrast, recent immunodetection experiments confirmed the presence of CRT in a fraction of loosely bound wall proteins that were released by the collagenase treatment of lupine, maize, and *Arabidopsis* cell walls (Luczak et al. 2014). The location of CRT at the cellular periphery in these species was also confirmed by immunolocalization at both the light and electron microscopy levels. The biological function of CRT in plant ECM is not known; however, the protein contribution in cell-to-cell communication and the regulation of the architecture of plasmodesmata and the cell wall via CRT’s Ca^2+^-binding/buffering capacity have been suggested.

The FA is a high specialized extracellular structure that is involved in pollen tube reception, the import of metabolites, and the export of the pollen tube attractants, and various proteins localize in the FA (see the review by Dresselhaus and Frankling-Tong 2013). A recent interesting observation is that both CRT and exchangeable Ca^2+^ were concentrated within the FA of the *Petunia* receptive synergid at the time of sperm cell deposition (Lenartowski et al. 2015). Our studies in *Hyacinthus* confirm the location of CRT in the FA both prior to pollination as well as during the late stage of the progamic phase when the structure of the synergid cell wall at the micropylar pole is subjected to clear rebuilding. During the progamic phase, synergids accumulate loosely bound Ca^2+^ not only in the cytoplasm but also in the FA (Chaubal and Reger 1992; Tian and Russel 1997; Iwano et al. 2012). In contrast, at the fertilization, the aforementioned Ca^2+^ oscillations occur in the cytosol of the receptive synergid (Sandaklie-Nikolova et al. 2007; Iwano et al. 2012; Denninger et al. 2014; Ngo et al. 2014). The accumulation of Ca^2+^ prior to the fusion of gametes seems to be correlated with the preparation of the female gametophyte to adopt the pollen tube and, consequently, the male gametes. However, the mechanism regulating the level and availability of exchangeable Ca^2+^ that can be released from the ECM is not known. The main deposit of Ca^2+^ in the cell wall is low-methyl-esterified HGs (Grant et al. 1973; Peaucelle et al. 2012). However, our previous studies in hyacinth demonstrated that during the progamic phase, the FA is almost completely devoid of Ca^2+^-associated HGs (Niedojadło et al. 2015), suggesting that HGs do not constitute a dynamic deposit of Ca^2+^ in the area of the FA prior to fertilization. It is, therefore, possible that during the period preceding the gamete fusion, this function is played by CRT. In contrast, after fertilization, we reported a drastic increase in the level of Ca^2+^-associated HGs in the wall of the fertilized egg cell and in the ECM of both synergids, including the area of the FA (Niedojadło et al. 2015). Therefore, it is possible that the rearrangement of ECM considering the composition of HGs and the content of stored Ca^2+^ is essential only during embryogenesis.

In summary, our studies have revealed diverse levels of CRT expression in cells of the female gametophyte of *Hyacinthus* prior to and after fertilization, which may be associated with the participation of this protein in double fertilization, polyspermy block, and the initiation of embryo and endosperm development. CRT, as a multifunctional and multicompartmental protein with respect to its Ca^2+^-binding/buffering specificity, seems to be a good candidate for the control of local Ca^2+^ concentrations within female gametophyte cells not only in the cytoplasm but also in the ECM. In light of our results and other authors’ data, we conclude that the regulation mechanism of an optimal Ca^2+^ environment in the embryo sac involving CRT seems to be universal in angiosperms. In addition, our previous (Niedojadło et al. 2015) and present studies suggest that two different mechanisms may be engaged in the regulation of the optimal exchangeable Ca^2+^ level during reproductive events in *Hyacinthus*: the first involving CRT and the second associated with HG rearrangement in the ECM.

### **Author contribution statement**

KN and EBK conceived and designed the experiments; KN and RL performed the experiments; KN, RL, and EBK analyzed the data; KN, RL, ML, and EBK wrote the paper.

## Abbreviations

BrU: Bromouridine
Ca^2+^: Calcium/calcium ions
CRT: Calreticulin
CRTs: CRT isoforms
CRT PAb: Polyclonal antibody against CRT
ER: Endoplasmic reticulum
HG: Homogalacturonan
ECM: Extracellular matrix
FISH: Fluorescence in situ hybridization
FA: Filiform apparatus
